# Effects of the Taste Substances and Metal Cations in Green Tea Infusion on the Turbidity of EGCG–Mucin Mixtures

**DOI:** 10.3390/foods13081172

**Published:** 2024-04-12

**Authors:** Longjie Xu, Qingqing Ye, Qingqing Cao, Yuyi Liu, Xinghui Li, Zhengquan Liu, Yushun Gong, Sheng Zhang, Junfeng Yin, Yongquan Xu

**Affiliations:** 1 Key Laboratory of Tea Biology and Resources Utilization, Tea Research Institute Chinese Academy of Agricultural Sciences, Hangzhou 310008, China; xulongje@tricaas.com (L.X.); YEQQabcd@163.com (Q.Y.); caoqingqing@tricaas.com (Q.C.); lyynky123@163.com (Y.L.); yinjf@tricaas.com (J.Y.); 2 Institute of Tea Science, Nanjing Agricultural University, Nanjing 210095, China; lxh@njau.edu.cn; 3 Shenzhen Xin Rong Yang Food Technology Co., Ltd., Shenzhen 518000, China; 4 Institute of Sericulture and Tea, Zhejiang Academy of Agricultural Sciences, Hangzhou 310021, China; liuzq0312@163.com; 5 Key Laboratory of Tea Science of Ministry of Education, Hunan Agriculture University, Changsha 410128, China; gongyushun@foxmail.com (Y.G.); zhangsheng@hunau.edu.cn (S.Z.)

**Keywords:** green tea, mucin, taste compounds, astringency, turbidity

## Abstract

Astringency has an important impact on the taste quality of tea infusion, a process which occurs when polyphenols complex with salivary proteins to form an impermeable membrane. (-)-Epigallocatechin gallate (EGCG) is the main astringent compound found in green tea and mucin is the main protein present in saliva. Determining the turbidity of EGCG–mucin mixtures is an effective method to quantify the astringency intensity of EGCG solutions. In this study, the effects of taste-related, substances present during green tea infusion, on the turbidity of EGCG–mucin mixtures was investigated under the reacting conditions of a pH value of 5.0, at 37 °C, and for 30 min. The results showed that epicatechins, caffeic acid, chlorogenic acid, and gallic acid reduced the turbidity of EGCG–mucin mixtures, while rutin increased turbidity. Metal ions increased the turbidity of EGCG–mucin mixtures. These can be arranged by effectiveness as Al^3+^ > K^+^ > Mg^2+^ > Ca^2+^. Caffeine, theanine, and sodium glutamate all decreased the turbidity values of EGCG–mucin mixtures, but sucrose had a weak effect. Further experiments confirmed that the turbidity of green tea infusion–mucin mixture indicated the astringent intensity of green tea infusion, and that the turbidity was significantly correlated with the contents of tea polyphenols and EGCG.

## 1. Introduction

The perception of oral astringency is complex. It is usually caused by the intake of polyphenol-rich foods and is mediated by the whole oral perception system. Studies have shown that the perception of oral astringency is based on the interaction between polyphenols and mucins. Tea polyphenols, accounting for 18~36% of tea, are the main astringent substances present during green tea infusion [1]. About 70~80% of tea polyphenols are composed of catechins. Eight catechins, including four epicatechins and four non-epicatechins, have been described well. Epicatechins are the main source of astringency in green tea infusion [2]. The intensity of the astringency of catechins at the same concentrations is as follows: (-)-epigallocatechin gallate (EGCG) > (-)-epicatechin gallate (ECG) > (-)-gallocatechin gallate (GCG) > catechin gallate (CG) > (-)-epigallocatechin (EGC) > (-)-epicatechin (EC) > catechin (C) > (+)-gallocatechin (GC). EGCG is the most important astringent substance in green tea infusion [3,4]. Besides catechins, some other polyphenols, such as phenolic acids (PAs) and rutin [3], are vital to the astringency of green tea infusion [5]. Green tea infusion not only causes an astringent taste, but also introduces elements of bitterness, umami, and sweetness. Catechins and caffeine are the main bitter compounds in tea infusion. Amino acids, such as theanine, glutamic acid, and aspartic acid, provide an umami taste for tea infusion, and the quality of tea is closely related to the content of these amino acids [6]. In addition to a small amount of sweet amino acids, soluble sugars are the main contributors to the sweet taste in tea infusion.

Sensory evaluation is an effective method with which to characterize the astringency intensity of tea infusion, but it is susceptible to the subjectivity of sensory evaluation panelists [7]. Astringency perception training and the establishment of corresponding sensory scales are required for panelists before they conduct sensory evaluations of astringency intensity, which are time-consuming and tedious. Currently, most studies depend on sensory evaluation to assess the astringency intensity of green tea infusion. Turbidity determination is considered to be the most intuitive method for studying insoluble aggregates in vitro and has been used to investigate the relationships between the structures of polyphenols and their affinity to mucin [8,9]. Our previous experiments [10] suggested that the turbidity value was correlated with the astringency intensity of polyphenols and an objective astringency intensity evaluation method was established based on it [9,11,12]. Several studies demonstrated that astringency was more closely related to the interactions between polyphenols and proteins compared to the precipitation between tannins and proteins, and the in vitro evaluation of the astringency intensity of polyphenols was reliable when conducted using the interactions between polyphenols and proteins [13,14,15]. However, the impact of other constituents present during green tea infusion on the turbidity determination remained unclear. In this study, typical constituents present in green tea infusion (epicatechins, PAs, rutin, caffeine, theanine, sodium glutamate, sucrose and metal ions) were added to the mucin solution and EGCG–mucin mixtures, and the changes in turbidity were monitored. Later, the correlation between the turbidity of a green tea infusion–mucin mixture and the astringent intensity of green tea infusion were investigated, and major contributors to the turbidity were screened out. The results provide evidence for the application of the turbidity method in order to objectively evaluate the astringency intensity of green tea infusion.

## 2. Materials and Methods

### 2.1. Materials

(-)-Epicatechin gallate (ECG, chromatographically pure) and (-)-epigallocatechin gallate (EGCG, chromatographically pure) were purchased from Taiyo Greenpower Co., Ltd. (Wuxi, China). Mucin (from the porcine stomach), (-)-epigallocatechin (EGC), (-)-epicatechin (EC), caffeic acid, chlorogenic acid, gallic acid and rutin were purchased from Sigma-Aldrich (Shanghai, China). Potassium chloride (KCl), calcium chloride (CaCl_2_), magnesium chloride (MgCl_2_) and aluminum chloride (AlCl_3_) were all analytically pure and were obtained from Sinopharm Chemical Reagent Co., Ltd. (Shanghai, China). We used a Hach TL2300 Turbidimeter (Shanghai, China). Tea samples were obtained from the Tea Research Institute, Chinese Academy of Agricultural Sciences (Hangzhou, China). The brewing water used was pure water obtained from Hangzhou Wahaha Group Co., Ltd. (Hangzhou, China).

### 2.2. Methods

#### 2.2.1. Determination of Turbidity Value

The turbidity value was measured using Hach TL2300 Turbidimeter (Shanghai, China) in NTU (Nephelometric Turbidity Unit). All samples were measured 6 times. Previous experiments carried out comparative analysis of the in vitro complexation model of a mucin monomer, combined with sensory analysis. The polyphenol concentration–astringency intensity model was established based on the turbidity value of the complexation reaction between polyphenols and mucin, as determined by the turbidity method. Then, we established the in vitro evaluation model of mucin turbidity–astringency intensity [10].

(1) $$\mathrm{Astringency}\;\mathrm{intensity}=-0.0004x^{2}+0.2036x-0.2538$$

where x means the turbidity in NTU values, R^2^ = 0.994.

#### 2.2.2. Study on the Complexation between Epicatechins and Mucin

According to the previous experimental results [10], the highest turbidity value was detected when the mucin interacted with EGCG at a pH of 5.0 at 37 °C, indicating that the complexation between mucin and polyphenols was exhibited to the greatest extent under the reaction conditions used. In this study, different concentrations of epicatechins were complexed with mucin solution to analyze the change in turbidity values. The concentrations of epicatechins were set at 0.65, 1.31, 1.96, and 2.62 mM. The turbidity value was measured using Hach TL2300 turbidity in NTU. All samples were repeatedly measured 6 times.

#### 2.2.3. Interaction Turbidity Value between Epicatechins

EGCG with concentrations of 0, 0.65, 1.31, 1.96 and 2.62 mM was mixed with EGCG (0.65 mM), ECG (0.28 mM), EGC (1.96 mM), and EC (2.07 mM) with an astringency intensity of 2 points. We then mixed the mucin (0.3%) solution with EGCG and epicatechins (V_EGCG+Epicatechins_:V_mucin_ = 1:1) at a pH 5.0 and at 37 °C for 30 min. The turbidity values of the mixed solution were measured in the same way as in Section 2.2.1.

#### 2.2.4. Interaction Turbidity Value between EGCG and Pas

Caffeic acid, chlorogenic acid, gallic acid and rutin solution were mixed with EGCG solution, respectively. We added EGCG with the same astringency intensity as PAs to different concentrations of EGCG as control groups (Table 1). Then, the mucin (0.3%) solution was mixed with EGCG and PAs (V_EGCG+PAs_:V_mucin_ = 1:1) under conditions of pH 5.0 and 37 °C for 30 min. The turbidity values of the mixed solution were measured in the same way as in Section 2.2.1.

#### 2.2.5. Interaction Turbidity Value between EGCG and Metal Cations (MCs)

EGCG was mixed at concentrations of 0, 0.65, 1.31, and 1.96 mM with 0, 0.51, 1.02, 1.53, 2.05, 2.56 mM of K^+^, 0, 0.125, 0.25, 0.50, 1.00, and 2.00 mM of Ca^2+^, 0, 0.08, 0.16, 0.33, 0.49, and 0.74 mM of Mg^2+^, and 0, 0.07, 0.15, 0.22, 0.30, and 0.37 mM of Al^3+^. Then, we mixed the mucin (0.3%) solution with EGCG and MCs (V_EGCG+MCs_:V_mucin_ = 1:1) under a pH of 5.0 at 37 °C for 30 min. The turbidity values of the mixture solution were measured in the same way as outlined in Section 2.2.1.

#### 2.2.6. Interaction Turbidity Value between EGCG and Typical Taste Substances (TTSs)

EGCG with concentrations of 0, 0.65, 1.31, and 1.96 mM was mixed with 1.25 and 1.75 mM of caffeine; 1.7 and 17.2 mM of theanine; 0.3, 0.6, 3.0, and 11.8 mM of sodium glutamate; and 0.3, 2.9, 14.6, and 58.4 mM of sucrose. Then, we mixed the mucin solution (0.3%) with EGCG and TTSs (V_EGCG+TTSs_:V_mucin_ = 1:1) under a pH of 5.0 at 37 °C for 30 min. The turbidity values of the mixed solution were measured in the same way as outlined in Section 2.2.1.

#### 2.2.7. Verification in Green Tea Infusion

The green tea was brewed using boiling water (m:V = 1:50) to induce tea infusion. The mucin solution (0.3%) was mixed during tea infusion (V_Tea infusion_:V_mucin_ = 1:1) under conditions of a pH value of 5.0 at 37 °C for 30 min. The turbidity values of the mixture solutions were measured in the same way as outlined in Section 2.2.1.

##### Detection of Main Astringent Substances in Green Tea Infusion

The main astringent substances present during green tea infusion were detected via HPLC with a UV detector (HPLC/UV; Shimadzu, Kyoto, Japan) and we used a C18 column known as Diamonsil™ (4.6 mm × 250 mm, 5 μm; Dikma Technologies Inc., Lake Forest, CA, USA) for the separation reaction, with the column’s temperature set at 35 °C. We prepared mobile phases A of water/2% (*v*/*v*) acetic acid and mobile phases B of 100% acetonitrile, respectively. The post-run time was 5 min. The injection volume was 10 μL. There was a flow rate of 1 mL/min and a detection wavelength 280 nm. The elution gradient began with 6.5% B, which was increased to 15% B at 16 min. This level was maintained until 25 min, when it finally back to 6.5% B at 30 min.

##### Sensory Evaluation

Sensory evaluation was carried out at room temperature in a special tea-tasting room. We took 3 g of tea leaves and added it to a tea cup with a 1:50 (m:V) ratio of tea to boiling water. The sensory evaluation of the tea infusion was conducted by a group of seven trained members (22–55 years old), consisting of three males and four females from the Tea Research Institute, Chinese Academy of Agricultural Sciences. All the sensory evaluation group members were trained to sip the tea infusion, retain it in the mouth for 8–10 s, and spit out it after scoring the astringency intensity relative to the astringency reference solution. To evaluate the green tea infusion samples, a sensory score was used on a 10-point scale as follows: 0–2 is “very weak”; 2–4 is “weak”; 4–6 is “neutral”; 6–8 is “strong”; and 8–10 “very strong”. After scoring each sample, the group members were asked to rinse their mouths with pure water. To reduce any possible legacy effects, group members were also asked to rest for 5 min after each tested sample to reduce taste fatigue.

### 2.3. Statistical Analysis

Microsoft Excel 2019 (Microsoft Corporation, Redmond, WA, USA) and SPSS Statistics 24 (IBM Corporation, Amunk, NY, USA) were utilized to conduct statistical data analysis. OriginPro 2023 (OriginLab Corporation, Northampton, MA, USA) was used to make graphs.

## 3. Results and Discussion

### 3.1. Effect of Epicatechins on the Turbidity of Mucin Solution and EGCG–Mucin Mixtures

**Figure 1:**
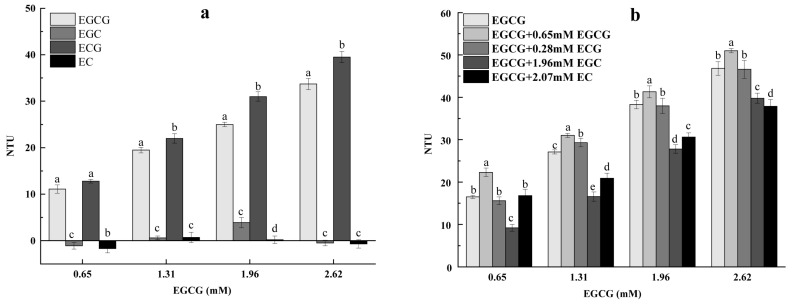
The interaction turbidity values of epicatechins with mucin and EGCG. (**a**) The interaction turbidity values between the same concentrations of epicatechins and the EGCG–mucin solution. (**b**) The interaction turbidity values between the same astringency intensity of epicatechins and the EGCG–mucin solution. (letters a–e *p* < 0.05).

As shown in Figure 1a, the significant addition of EGCG and ECG in a concentration-dependent manner increased the turbidity of the mucin solution. However, the addition of certain concentrations (0.65 and 2.62 mM) of EGC and EC decreased the turbidity of the mucin solution. At the same molar concentration, the turbidity values of different epicatechin–mucin mixtures were in the order of ECG > EGCG > EC > EGC. This was not fully consistent with the previous findings showing that polyphenols with higher molecular weights had higher affinities to proteins [8]. A previous study demonstrated that the astringency intensity of epicatechins at the same molar concentration was ECG > EGCG > EGC > EC. There was a positive correlation between the turbidity of epicatechin–mucin mixtures and the astringency intensity of epicatechins.

According to the results of sensory evaluation, epicatechins had additive effects on astringency [8]. In this study, the effects of epicatechins on the turbidity of EGCG–mucin mixtures were analyzed. As shown in Figure 1b, the effects were not simply additive. Four epicatechins, each with the same astringency intensity, caused significantly different turbidity changes in EGCG–mucin mixtures. The turbidity value of the EGCG–mucin solution increased with the addition of EGCG, was unchanged with the addition of ECG, and decreased with the addition of EGC and EC (Figure 1b). The changing trends were consistent with the effects of each epicatechin on the turbidity of mucin solutions, except ECG (Figure 1a). The underlying mechanism was unclear. In summary, the results showed that the effects of four epicatechins on the turbidity of the mucin solution and EGCG–mucin mixtures were different. The turbidity determination results showed that there was an inhibitory effect between epicatechins, which was inconsistent with the results of the sensory evaluation [5].

### 3.2. Effect of PAs on the Turbidity of Mucin Solution and EGCG–Mucin Mixtures

#### 3.2.1. Caffeic Acid

**Figure 2:**
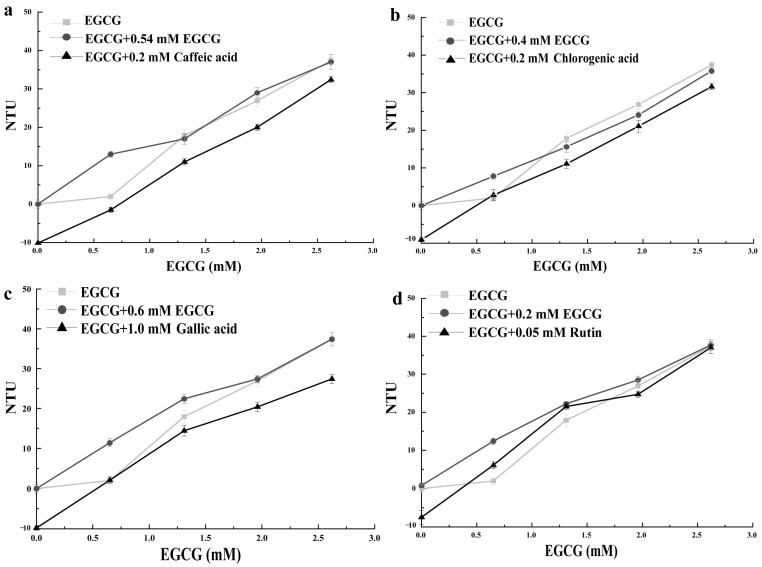
Effect of caffeic acid (**a**), chlorogenic acid (**b**), gallic acid (**c**), and rutin (**d**) on the turbidity values of EGCG–mucin interactions at a pH of 5.0 and at 37 °C when incubated for 30 min.

Caffeic acid has an astringent taste [16]. It presents a sour taste at high concentrations [5]. The intensities of both taste attributes are strengthened with the increase in concentrations. Caffeic acid decreased the turbidity of the mucin solution (Figure 2a) and EGCG–mucin mixtures, suggesting an astringency inhibition effect. However, the inhibition of the turbidity of mucin solution was weakened with the increasing concentrations of EGCG [5]. Compared with the addition of EGCG, the turbidity of the EGCG–mucin mixture was lower after the addition of caffeic acid with the same predicted intensity of astringency.

#### 3.2.2. Chlorogenic Acid

Chlorogenic acid tastes slightly astringent [17]. It presents a sour taste at high concentrations. The intensities of both tastes were enhanced with the increase in concentrations. As shown in Figure 2b, chlorogenic acid reduced the turbidity value of mucin solution and EGCG–mucin mixtures. Compared with the addition of EGCG, the turbidity of EGCG–mucin mixture was generally lower after the addition of chlorogenic acid with the same predicted intensity of astringency. The decrease in turbidity values indicated that chlorogenic acid had an astringency inhibition effect, which it probably exerted by weakening the complexation between EGCG and mucin. Chlorogenic acid affected the functional properties of EGCG–mucin mixture by changing the structure of mucin [18].

#### 3.2.3. Gallic Acid

The taste of gallic acid is similar to that of caffeic acid [19]. The effect of gallic acid on the turbidity of mucin solution and EGCG–mucin mixtures is also similar to that of caffeic acid (Figure 2c). In our study, it decreased the turbidity of the mucin solution and EGCG-mixtures. Compared with the addition of EGCG, the turbidity of the EGCG–mucin mixture was significantly lower after the addition of gallic acid with the same predicted intensity of astringency. This suggested that gallic acid reduced the affinity of EGCG and mucin in the EGCG–mucin mixture solution and lowered the astringency of EGCG.

#### 3.2.4. Rutin

Rutin is a representative astringent compound. It reduced the turbidity value of the mucin solution (Figure 2d). The effect of rutin on the turbidity of EGCG–mucin mixtures was fluctuant. Unlike when EGCG alone is added to PAs, the turbidity of the EGCG–mucin mixture was significantly higher after the addition of rutin with the same predicted intensity of astringency. This suggested that rutin had an additive effect on the astringency of EGCG [5,20].

### 3.3. Effect of MCs on the Turbidity of Mucin Solution and EGCG–Mucin Mixtures

#### 3.3.1. Potassium Ion (K^+^)

**Figure 3:**
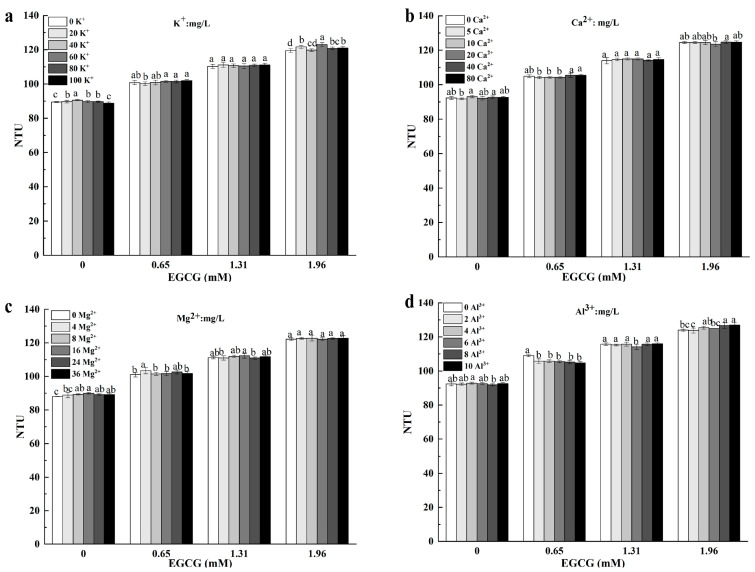
Effect of K^+^ (**a**), Ca^2+^ (**b**), Mg^2+^ (**c**) and Al^3+^ (**d**) on turbidity value of EGCG–mucin interaction at a pH 5.0 at 37 °C when incubated for 30 min. (letters a–d *p* < 0.05).

K^+^ tastes astringent but slightly sweet. The turbidity value of the mucin–K^+^ solution was greater than 80 NTU (Figure 3a), indicating that K^+^ increased the turbidity value of the mucin solution. When the concentrations of K^+^ ranged from 0 to 1.02 mM, the turbidity of the mucin solution increased as the concentrations of K^+^ increased. When the concentrations of K^+^ ranged from 1.02 to 2.56 mM, the turbidity of the mucin solution decreased as the concentrations of K^+^ increased. The turbidity value reached its maximum when the K^+^ concentration was 1.02 mM. When the concentration of EGCG was 1.96 mM in the EGCG–mucin mixture, the addition of K^+^ significantly increased the turbidity of the EGCG–mucin mixture, indicating that K^+^ promoted the complexation between EGCG and mucin. Microscopic characterization is needed to prove this.

#### 3.3.2. Calcium Ion (Ca^2+^)

The main taste attribute of Ca^2+^ is astringency. When at high concentrations, Ca^2+^ is slightly salty. When the concentration of Ca^2+^ was higher than 4 mg/L, there was bitter and astringent taste in the tea infusion [21]. Ca^2+^ significantly increased the astringency intensity of EGCG [22,23] in a concentration-dependent manner. Ca^2+^ mainly increased the astringency of tea infusion by complexing with tea polyphenols [24]. The addition of Ca^2+^ enhanced the astringency of catechins by enhancing its binding ability to oral salivary mucins in the oral cavity, which ultimately strengthened the oral astringency caused by catechins [21]. The addition of Ca^2+^ hardly changed the turbidity of mucin solutions and EGCG–mucin solutions (Figure 3b). Hu et al. (2014) proved that the effect of Ca^2+^ on the precipitation of tea infusion was positively correlated with the concentration of Ca^2+^. The ratio of polyphenols in tea infusion also affected the formation of precipitation [25]. Ca^2+^ can interact with other salivary proteins or tea constituents to enhance the astringency of green tea infusion.

#### 3.3.3. Magnesium Ion (Mg^2+^)

The taste characteristics of Mg^2+^ are similar to those of Ca^2+^. Unlike Ca^2+^, Mg^2+^ mainly causes astringency and numbness of the tongue [26]. At low concentrations, Mg^2+^ has weak saltiness and produces an obvious sense of exogenous substances, which is different from the taste of pure water. The presence of Mg^2+^ attenuated the taste of cold-water-soluble instant green tea infusions, decreasing the quality [27]. When the concentrations of Mg^2+^ ranged from 0 to 0.33 mM, the turbidity of the mucin solution increased as the concentrations of Mg^2+^ increased. When the concentrations of Mg^2+^ ranged from 0.33 to 0.74 mM, the turbidity of mucin solution remained similar while the concentrations of Mg^2+^ increased. The turbidity value reached its maximum value when the Mg^2+^ concentration was 0.33 mM (Figure 3c). When the concentration of EGCG in the ECG-mixture was 1.96 mM, the turbidity value of EGCG–mucin mixture was not altered after the addition of Mg^2+^. This implied that the Mg^2+^ had no significant effect on the complexation of EGCG and mucin and the astringency intensity of tea infusion.

#### 3.3.4. Aluminum Ion (Al^3+^)

The astringency of Al^3+^ is similar to the taste of unripe persimmon. It was reported that catechins present during tea infusion were complexed with Al^3+^ at a ratio of 1:1 [28]. Al^3+^ had a weak influence on the turbidity values of mucin solutions (Figure 3d). The effect of Al^3+^ on the EGCG–mucin mixtures depended on the EGCG concentration. When the concentration of EGCG was 0.65 mM, Al^3+^ distinctly reduced the turbidity value. When the concentration of EGCG was 1.96 mM, the turbidity of the mixture significantly increased as the concentration of Al^3+^ increased. MCs changed the secondary structures of mucins, affecting the conformation of proteins. This, in turn, led to changes in hydrophobicity [29]. Interactions between MCs and the functional groups of mucin also affected the complexation, changing the taste characteristics of tea infusion. This was probably due to the conformational changes of mucin induced by metal ions [30]. It is not recommended to brew green tea with water that contains high levels of Al^3+^ as it may increase the taste of astringency.

### 3.4. Effect of TTSs in Tea Infusion on the Turbidity of Mucin Solution and EGCG–Mucin Mixtures

#### 3.4.1. Caffeine

**Figure 4:**
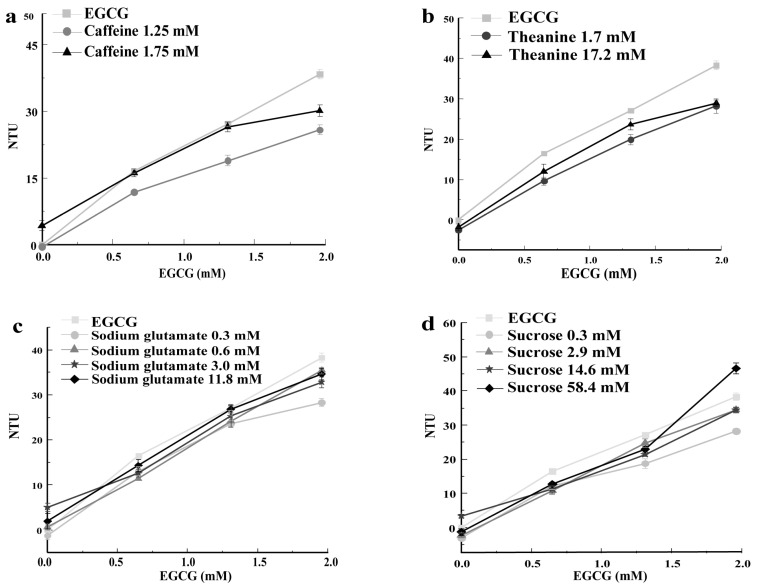
Effect of caffeine (**a**), theanine (**b**), sodium glutamate (**c**), and sucrose (**d**) on the interaction turbidity values of a EGCG–mucin solution at a pH of 5.0 at 37 °C when incubated for 30 min.

Caffeine is an important source of bitter taste in tea infusion [22,31]. Meanwhile, caffeine interacts with other taste compounds to modify the taste of green tea infusions [3]. As shown in Figure 4a, 1.75 mM of caffeine reduced the turbidity value of the EGCG–mucin solution. Previous studies demonstrated that caffeine and catechins tended to form complexes via hydrogen bonds. This prevented the binding of catechins and salivary proteins, and changed the taste characteristics of tea infusion by increasing the umami taste and reducing the bitterness [23]. Brannan et al. (2001) found that the astringency of alum and tannins remained stabled or slightly decreased after the addition of citric acid, sucrose, caffeine, and sodium chloride [32]. In this study, caffeine reduced the turbidity values of EGCG–mucin mixtures, providing more evidence that the interaction between caffeine and EGCG reduced the astringency intensity of EGCG.

#### 3.4.2. Theanine

Theanine presents a slight umami and sweet taste at high concentrations [23]. Yin et al. (2014) proved that EGCG reduced the umami taste of theanine, while theanine enhanced the bitterness and reduced the astringency of EGCG [21]. Theanine decreased the turbidity of mucin solutions and EGCG–mucin mixtures, indicating that theanine reduced the astringency intensity. According to the results (Figure 4b), theanine showed a stronger effect in terms of decreasing the turbidity at a lower concentration. Liu et al. (2023) demonstrated that umami amino acids inhibited the astringent taste presentation of catechins [33].

#### 3.4.3. Sodium Glutamate

Sodium glutamate is an umami compound [34]. As shown in Figure 4c, the effect of sodium glutamate on the turbidity values of the mucin solution and EGCG–mucin mixtures was concentration-dependent. The effect of sodium glutamate in terms of decreasing the turbidity was more obvious when the mixtures contained higher levels of EGCG. Low concentrations of sodium glutamate were more effective in decreasing the turbidity of EGCG–mucin mixtures. Among test concentrations, the one 0.3 mM of sodium glutamate was the most effective. It was found that sodium glutamate, caffeine, and theanine exhibited similar effects on the turbidity values of EGCG–mucin mixtures.

#### 3.4.4. Sucrose

Sucrose is a sweet substance present in green tea infusions [22]. One study showed that the presence of sucrose had no significant effect on the astringency intensity of EGCG, except that the highest test concentration of sucrose (54.8 mM) had a slight inhibitory effect on the astringency intensity of EGCG [12]. Some other studies showed that EGCG reduced the sweetness of sucrose and that sucrose reduced the bitterness and astringency of EGCG [21,32]. In this study, sucrose had a weak effect on the turbidity of mucin solution and a decreasing effect on the turbidity of EGCG–mucin mixtures (Figure 4d). The effect was more obvious as the concentrations of EGCG increased in the EGCG–mucin mixtures. Interestingly, the addition of 58.4 mM of sucrose to the EGCG (1.96 mM)–mucin mixture significantly increased its turbidity.

### 3.5. Astringency Intensity Evaluation of Green Tea Infusion by the Turbidity Method

#### 3.5.1. Analyses of the Main Astringent Substances in Green Tea Infusion

**Figure 5:**
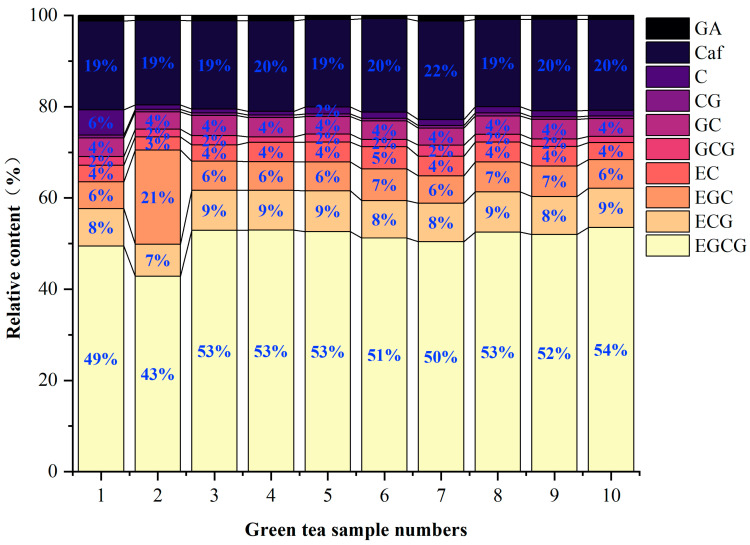
Relative content analyses of the main astringent substances in green tea infusion. The green tea samples were numbered 1~10.

The relative content of the main astringent substances in 10 green tea infusions is shown in Figure 5. The relative content of each astringent-tasting substance in the 10 green tea infusion samples was similar. EGCG and caffeine were major astringent compounds. EGCG had the highest content (43–54%), followed by caffeine (19–22%). An exception is tea infusion 2, in which the relative content of EGC (21%) was higher than that of caffeine, clearly distinguishing it from the other green tea infusion samples.

#### 3.5.2. Correlation Analyses between Turbidity Value and Astringency Intensity of Green tea Infusion

**Figure 6:**
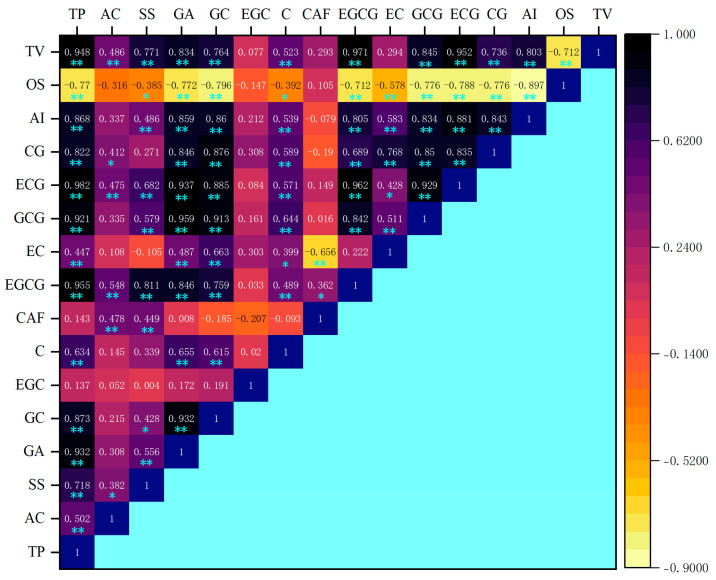
Correlation analysis between turbidity values and the astringency intensity of green tea infusion and mucin reaction (**—significant correlation at the *p* < 0.01. *—significant correlation at the *p* < 0.05.) (Tea polyphenol (TP), amino acid (AC), soluble sugar (SS), gallic acid (GA), gallocatechin (GC), epigallocatechin (EGC), catechin (C), caffeine (CAF), epigallocatechin gallate (EGCG), epicatechin (EC), gallocatechin gallate (GCG), epicatechin gallate (ECG), catechin gallate (CG), astringency intensity (AI), overall score (OS), turbidity value (TV)).

In this study, the turbidity determination of green tea infusion–mucin mixtures and the sensory evaluation of the astringency intensity of green tea infusion were carried out. The correlations between astringency intensity, overall taste score, turbidity, and the relative content of taste substances are shown in Figure 6. A negative correlation between astringency intensity and overall taste score was observed, suggesting astringency had an adverse effect on the taste quality of green tea infusion. The relative contents of most taste substances, except amino acids, EGC, and caffeine, were significantly correlated with the astringency intensity of green tea infusion (*p* < 0.01). This was consistent with the sensory evaluation results in terms of the astringency intensity of corresponding taste substances. The correlations between the turbidity of green tea infusion–mucin mixture and the relative content of polyphenols and EGCG were higher than those of others, indicating that polyphenols and EGCG had the most significant (** 0.955) effect on the turbidity of green tea infusion–mucin mixtures. Other taste substances, except EGC, caffeine, and EC, also showed a significant correlation with the turbidity (*p* < 0.01). The turbidity of green tea infusion–mucin mixture was strongly and positively related to astringency intensity of green tea infusion, suggesting that it was possible to use turbidity determination to evaluate astringency intensity.

## 4. Conclusions

The four epicatechins examined had different effects on the turbidity of EGCG–mucin mixtures. EGCG and ECG increased turbidity, while EGC and EC decreased turbidity. The effects of EGC and EC were more obvious in EGCG–mucin mixtures with higher levels of EGCG. Caffeic acid, chlorogenic acid, and gallic acid reduced the turbidity values of EGCG–mucin mixtures. On the contrary, rutin increased turbidity. MCs affected the taste of tea infusion by complexing with mucin. The ability of MCs to increase the turbidity of EGCG–mucin mixtures was ranked as Al^3+^ > K^+^ > Mg^2+^ > Ca^2+^. Meanwhile, the sensory evaluation for metal ions still needs to be standardized. Caffeine, theanine, and sodium glutamate were all observed to lower the turbidity values of EGCG–mucin mixtures, and their effects will help to improve the taste of green tea infusion. Sucrose still needs further study to assess the negative regulation effect of astringency in green tea infusion. A strong correlation was observed between turbidity values and actual astringency intensity of green tea infusion. The possible explanation was that the astringency formation mechanism of green tea infusion was more complex than the interaction between taste substances and mucin. Further research focusing on the multi-interactions between oral saliva and green tea infusion is needed.

## Figures and Tables

**Table 1:** The EGCG concentration corresponds to the concentration of PAs under the same astringency intensity.

PAs/0.2 mM	Equal Astringency Intensity/Scores	EGCG/mM
Caffeic acid	1.5	0.5
Chlorogenic acid	1.0	0.4
Gallic acid	1.8	0.6
Rutin	0.5	0.2

## Data Availability

The original contributions presented in the study are included in the article, further inquiries can be directed to the corresponding author.
